# Chemotactic Sensing towards Ambient and Secreted Attractant Drives Collective Behaviour of *E. coli*

**DOI:** 10.1371/journal.pone.0074878

**Published:** 2013-10-03

**Authors:** Tine Curk, Davide Marenduzzo, Jure Dobnikar

**Affiliations:** 1 Department of Chemistry, University of Cambridge, Cambridge, United Kingdom; 2 Department of Physics, University of Edinburgh, Edinburgh, United Kingdom; 3 Department of Theoretical Physics, Jožef Stefan Institute, Ljubljana, Slovenia; University of Sheffield, United Kingdom

## Abstract

We simulate the dynamics of a suspension of bacterial swimmers, which chemotactically sense gradients in either ambient or self-secreted attractants (e.g. nutrient or aspartate respectively), or in both. Unlike previous mean field models based on a set of continuum partial differential equations, our model resolves single swimmers and therefore incorporates stochasticity and effects due to fluctuations in the bacterial density field. The algorithm we use is simple enough that we can follow the evolution of colonies of up to over a million bacteria for timescales relevant to pattern formation for *E. coli* growing in semisolid medium such as agar, or in confined geometries. Our results confirm previous mean field results that the patterns observed experimentally can be reproduced with a model incorporating chemoattractant secretion, chemotaxis (towards gradients in the chemoattractant field), and bacterial reproduction. They also suggest that further experiments with bacterial strains chemotactically moving up both nutrient and secreted attractant field may yield yet more dynamical patterns.

## Introduction

Microbial colonies such as suspensions of bacteria can form striking and spectacular patterns, whether in nature, or in the lab on a *Petri* dish. Examples are provided by biofilms and microbial mats [1]–[3], or by the regular or amorphous clusters and ring patterns formed by motile *E. coli* and *S. typhimurium* cells which are grown on dilute agarose gel [4]–[7], or by bacterial suspensions in microfluidic chambers and inside mazes [8]. The latter patterns are particularly relevant to our work here: because they form in a controlled environment in the lab, one may imagine that the mechanism underlying their arousal should be relatively simple. Indeed, a lot of models have been proposed in the literature [9]–[13] to account for the formation of microbial rings, whether swarming or static, and spots. Such models have established that these patterns can result from collective behaviour driven by interactions between the bacteria, such as chemotactic aggregation [7], competition for food [14], or even solely through a combination of reproduction and a density-dependent swim speed [15].

Almost invariably, the models proposed in the literature to study bacterial pattern formation in semisolid media involve the study of a system of partial differential equations which follow the coupled dynamics of the bacterial population, and, when needed, of a set of chemicals which affect bacterial motility. Therefore, the resulting description is a mean field one, where fluctuations are generally disregarded. In our work, instead, we take a different approach, and set up a microscopic model which follows the evolution of individual bacteria, whose motility is coupled to a set of continuum equations to model nutrient diffusion and consumption, as well as chemoattractant production, or secretion. This framework naturally includes noise and can model fluctuations in the bacterial density and their associated effects. This may be particularly important when pattern formation arises as a non-equilibrium phase transition between a uniform and a pattern-forming phase [7], [15]–[17] (this is the case in most theoretical models proposed thus far).

Resolving individual bacteria is computationally challenging, as the timescales relevant to pattern formation in bacterial colonies in agar are of several hours, and within this time bacteria grow so that their number can increase essentially exponentially in time. In view of these difficulties, we refrained from modelling the chemotaxis at the molecular level [18] and rather designed a simple and fast algorithm, where chemotaxis is modelled simply by a drift velocity in the direction of concentration gradients, and is coupled to a stochastic rule for bacterial swimming and replication, as well as to deterministic rules for nutrient consumption and secretion of chemoattractant. Via large scale parallel simulations, we show here that we can easily reach colony size and timescales of respectively millions of bacteria and several hours, which are well in the experimentally relevant range.

Our main result is that our microscopic model can account for a wide range of bacterial patterns previously observed experimentally, either in microfluidic chambers, or on a *Petri* dish. In particular, we first show that chemotactic bacteria spontaneously accumulate inside narrow boxes, and aggregate into clusters whose spatial position can be controlled by the confining geometry. We also reproduce the dynamic and steady-state patterns in agarose gel reported in Refs. [4], [5], where a small inoculum spreads out as a swarming ring, leaving behind an array of microbial spots. Another important result is that in our computer simulations we can vary the relative chemotactic sensitivity to nutrient and to secreted chemoattractant gradients: if the ratio between the former and the latter becomes large enough, then the bacterial spots become motile and the eventual pattern is a ring made up or merging moving spots rather than an amorphous or regular array of static ones. We hope that these results will spur further experimental work on bacterial strains with controlled sensitivity to nutrient and chemoattractant fields.

In our model, the essential mechanism leading to pattern formation is a self-trapping feedback mechanism: bacteria can accumulate locally due to, for instance, a density fluctuation, this triggers an increase in the local chemoattractant secretion, which leads via chemotaxis to even more bacteria in that spot, etc. This explanation also suggests that density fluctuations, which our model correctly captures, can be important in practice, as, for instance, they will affect the parameter values required for the onset of pattern formation.

Before presenting our results, a note on the nomenclature. In what follows we will refer to the secreted attractant as the ‘chemoattractant’ for simplicity, even though the nutrient field is also an ambient attractant for bacteria.

## Results

### Chemoattractant and geometry: spot formation

To begin with, we consider the case of an initially uniform suspension of motile bacteria which secrete chemoattractant at a constant rate 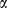. As mentioned in the Methods section, the bacteria interact with the chemoattractant concentration 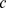 by adding a drift velocity which is proportional to the gradient of 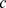.

**Figure 1:**
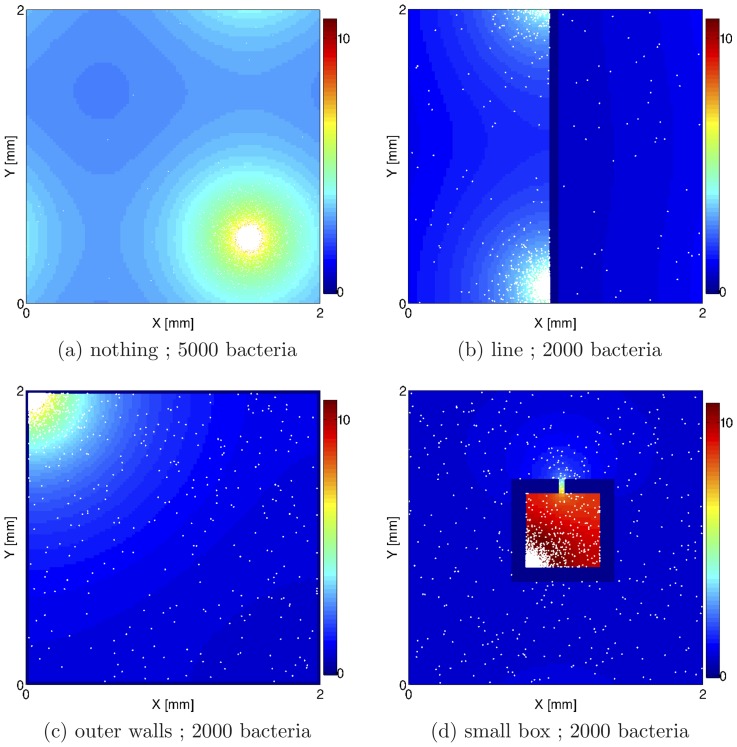
Resulting configurations after 3 Bacteria are white dots, chemoattractant field is represented with colours. Periodic boundary conditions were employed. Geometries are: (**a**) no boundary; (**b**) a wall; (**c**) four walls; (**d**) a box. Bacteria squeeze spontaneously into the small box in (d), just as they do in experiments realised with a similar geometry [8]. Parameters were: 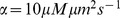, 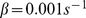, chemotactic efficiency 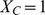. The chemoattractant concentration is presented by a colour code as displayed at the side of each figure in units of 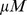: 0 (blue) to 10 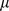
M (red).

Fig. 1A shows the dynamics observed in the absence of any boundaries. Here bacteria accumulate through a self-trapping feedback mechanism, which works as follows. Imagine that via a density fluctuations more bacteria occupy a region of space; as they are constantly producing chemoattractant, they will soon set up a chemoattractant source which attracts more bacteria, leading to further density increase, and to a positive feedback which enhances clustering. Apart from the diffusion of bacteria, the only force opposing the build-up of density is excluded volume, which sets the local concentration we observe in the cluster. Note that a similar self-trapping mechanism has been proposed for bacteria with density-dependent swim speed [15]; we will return to this simpler model later on. In all our simulations, the bacterial spots coarsen to yield a single cluster in a steady state.

While the tendency to aggregate is observed in an open geometry as shown in Fig. 1, the presence of boundary can localise the bacterial clusters, as shown in Figs. 1B–1D. Fig. 1B shows that when a wall is present the cluster sits there. Self-propelled particles have a known tendency to localise at walls when the time they need to get there is smaller (or much smaller) than the time associated with their rotational diffusion (or tumbling) [19], [20]. However, in our case the mechanism leading to the effective attraction to the wall is different: this is due to the fact that gradients of chemoattractant at the wall are larger (by about a factor of 2) than in the bulk, and as a consequence the aggregation tendency will be larger close to a boundary.

More complicated geometry can further control the location of the emerging patterns: for instance adding a corner localises the bacterial spot there, again due to the larger driving chemical gradient which can be created (Fig. 1C). Fig. 1D shows what happens if we place a square box in our simulation domain, with an opening on one of its sides (width 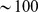 times larger than the size of a bacterium). This geometry resembles the one studied experimentally in Ref. [8], where it was found that chemotactic strains of bacteria spontaneously occupy the region within the box. Here our simulations reproduce this observations, and the mechanism is once more the coupling between production of chemoattractant and biased propulsion (chemotaxis). Our model also predicts that, just as in Fig. 1C, also within the box bacteria should accumulate at the corner. It would be interesting to check experimentally this prediction using larger boxes and high resolution microscopy experiments.

### Chemotaxis towards nutrient or towards secreted attractant: swarming rings and spots

In the previous Section we studied pattern formation starting from a uniform bacterial suspensions; here we want to instead study the dynamics starting from a localised inoculum of bacteria, which is more typical in experiments performed in semisolid media, such as dilute agarose gel where bacteria can still swim.

**Figure 2:**
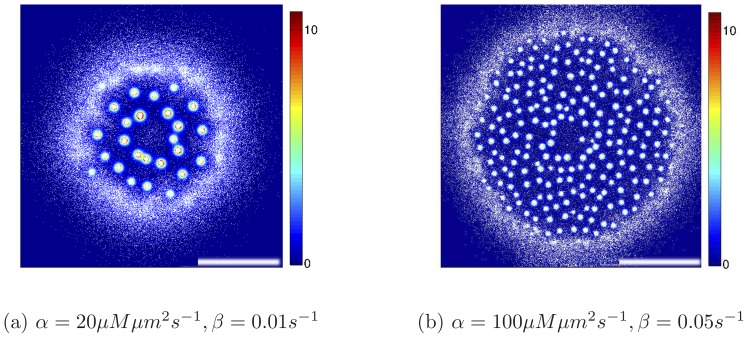
Patterns formed for a bacterial strain secreting chemoattractant, and initialised as a localised inoculum with 3 The scale bar is 1: 0 (blue) to 10 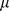
M (red). The population has a doughnut shape and moves outwards, and the spots left behind are stationary. The evolution has been followed here for a period corresponding to 4 hours of real time. Eventually the spots are unstable against coarsening and the final pattern, at very long time, should only feature a single cluster.

Fig. 2 shows the bacterial patterns formed over time as the inoculum spreads on a surface. Note that in the simulations in Fig. 2, the motile bacteria reproduce – this is important as the timescales typically probed in experiments with semi-solid medium span several bacterial generations. There is also nutrient in the simulations, which in Fig. 2 only controls the replication rate, according to a Michaelis-Menten-like law (see Methods).

Our simulations show that the bacterial population moves outwards. The outward motion resembles the formation of a Fisher wave [7], which forms in any microbial suspensions where the components are motile (i.e. diffuse) and replicate. In our case, however, the spreading wave has the shape of a ring, rather than a disk as is common for a Fisher-wave, and this is due to the interaction mediated by the chemoattractant. The spreading wave velocity is 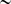 cm/hr, which is of the same order as a Fisher wave velocity in a medium where growth is rapid.

**Figure 3:**
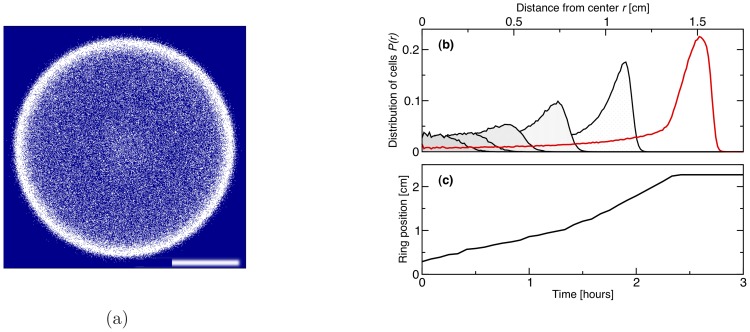
Evolution of a bacterial strain sensing only nutrients without secreting chemoattractant. (**a**) Pattern formation for a bacterial strain which is initialised as an 3 mm inoculum at the centre of the simulation domain, and is capable of chemotaxis only towards nutrient gradient: a swarming ring forms and moves out radially. The picture shows a snapshot obtained after 2 hours (real time). The scale bar is 1 cm. (**b**) The evolution of the radial distribution of the bacteria around the inoculum during the formation and expansion of the ring. The curves from the left to the right are the distributions after each 20 min of the simulation. The right-most solid red curve corresponds to the snapshot (a) after 2 hours. (**c**) The position of the ring as a function of time. The plateau is due to the final system size.

**Figure 4:**
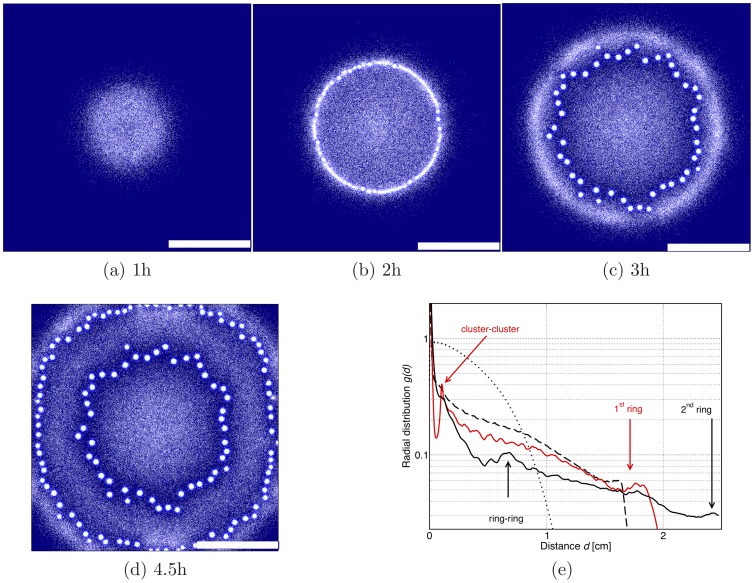
Patterns formed by a growing bacterial colony chemotactically sensing both chemoattractant and nutrient gradients. The scale bar is 1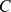 and is the same as in Figs. 1 and 2. Initially bacteria just grow and multiply (**a**), after some time their density is high enough that a ring first appears (**b**), which then breaks into spots (**c**). Spots lock spatially, while some bacteria escape from them and start spreading radially again; this leads to the formation and breakup of a further ring (**d**). The process would presumably continue indefinitely in an infinite domain. Parameters were 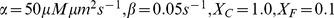. (**e**) Corresponding radial distribution functions for the four snapshots (dotted line (a), dashed line (b), solid red line (c), and solid black line (d)) with peaks at the characteristic length-scales in the system.

**Figure 5:**
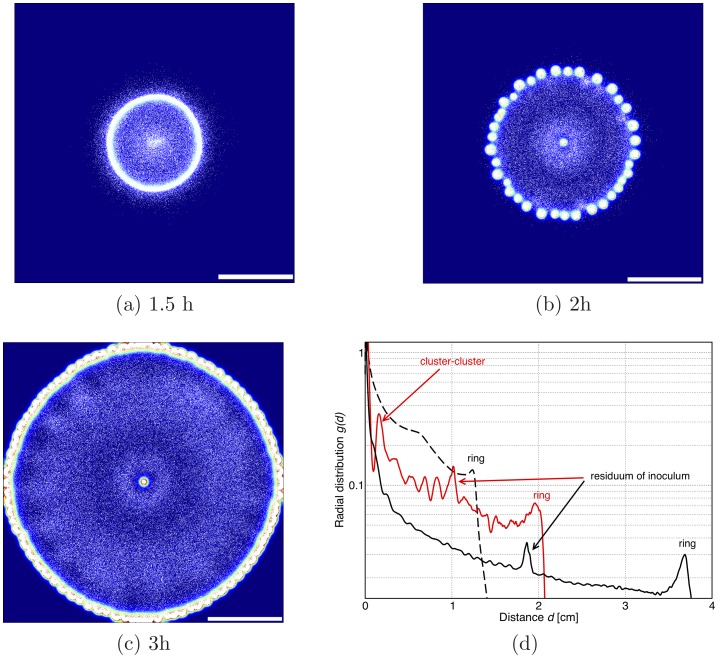
Dynamical patterns formed by a bacterial colony which chemotactically senses gradients in both chemoattractant and nutrient concentrations. The scale bar is 1(**a**) 1.5 h, (**b**) 2 h and (**c**) 3 h (real time). First a sharp ring forms, which then breaks into spots. After that spots move outwards and new spots grow until density is so high that spots practically again merge together in a ring. Parameters were 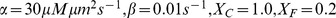. (**e**) Corresponding radial distribution functions for the three snapshots (dashed line (a), red solid line (b) and black solid line (c)) with peaks at the characteristic length-scales in the system.

The patterns in Fig. 2 resemble those observed in experiments with *E. coli* [4], [5], where the bacteria were observed to form a swarming ring depositing static bacterial droplets on its wake. While the stability of the final clustered structure only relies on the self-trapping mechanism identified when discussing the results in Fig. 1, and can therefore be explained by a variety of models [15], the correspondence between the transient patterns in experiments and simulations (see Figs. 2, 3, 4, 5) can only be achieved if nutrient-dependent growth rate and chemotaxis towards chemoattractant sources are both incorporated in the simulations.

While the strains simulated in Figs. 1 and 2 move up the gradient of chemoattractant, their drift speed is unaffected by the concentration field of nutrients. On the other hand, most bacterial strains do sense nutrient gradients as well as, or instead of, chemoattractant gradients. The chemotaxis towards nutrient gradients is actually an important, and very well studied, phenomenon in bacterial colonies, and we therefore asked whether we could reproduce experimental observations under those conditions as well. Therefore Fig. 3 shows the dynamics of a bacterial inoculum moving in a solution where nutrient is initially constant. As the bacteria consume the nutrient in the centre of the simulation domain, they sense the gradients which has been generated and move outwards, to reach higher nutrient concentration. In doing so, they form a swarming ring, which again resembles the one found on a Petri dish for strains which are only chemotactic towards a nutrient.

### Chemotaxis towards nutrient and secreted attractant: swarming rings and motile spots

Having explored the patterns observed for strains which sense chemotactically *either* a gradient in the nutrient or in the chemoattractant density, it is natural to ask for colonies which can sense both. This can on one hand be used to design further experiments to test our microscopic chemotactic model; it might also be directly relevant to existing experiments probing chemoattractant sensing, as it is difficult to completely eliminate chemotaxis towards nutrient sources.

Figs. 4 and 5 show the patterns observed when the chemotactic sensing is increased gradually. When the sensitivity to nutrient gradients is 10% of its maximum experimentally observed value, then the pattern in Fig. 2 becomes more ordered, as can be seen from Fig. 4. The higher symmetry in the pattern arises as the positions of the spots is guided by the swarming ring, which is more sharply focused when bacteria move chemotactically towards higher nutrient concentrations. In Fig. 4, we find that the swarming ring leads to the formation of spots arranged in two concentric rings – if the simulation were to proceed, we would expect the formation of further rings.

The higher symmetry in the spot position is to some extent reminiscent of the radially or hexagonally ordered patterns observed in some cases in experiments bacterial [4], [5], although the experimental patterns are more regular – possibly a better match would be found by considering larger samples and a larger number of bacteria in the spots, which would reduce stochasticity and disorder in the patterning.

As the efficiency of nutrient sensing is tuned up, the dynamic pattern changes quite dramatically. For instance, in Fig. 5 we show that when the sensitivity to nutrient gradients is doubled, spots no longer form in the wake of the advancing ring. Rather, the ring first expands, then breaks up into spots; however, these are still sufficiently motile up the nutrient gradient that they cannot be left behind. Due to replication, the microbial density in the moving ring is always increasing, this leads to more and more motile spots, which eventually merge once again. Both the dynamics and the steady-state patterns are therefore much affected by the ratio between the efficiency of chemotaxis to nutrient or chemoattractant gradients. Our results suggest that it would be interesting to perform systematic experiments with a series of different bacterial strains to identify the transition (or crossover) between dynamical patterns which we observed in the simulations in Figs. 2, 4 and 5.

### A comparison with the patterns found with density-dependent motility

It is instructive to compare the patterns found due to the chemotactic self-trapping in Figs. 1 and 2 with other spot patterns which can be found with similar model. For instance, it has been recently proposed that if the swim speed of a self-propelled particle decays with density, this can, if the decay is steep enough, lead to spot formation and phase separation [15]. The reason for pattern formation in that case is that bacteria (or indeed self-propelled particles), unlike passive particles, accumulate where they go slower. It is then possible to setup a self-trapping positive feedback loop similar to the one described for Fig. 1: due to fluctuations the local bacterial density increases; then this leads to further increase of the bacterial density (as bacteria accumulate where they are slow), which leads to further slowing down etc.

**Figure 6:**
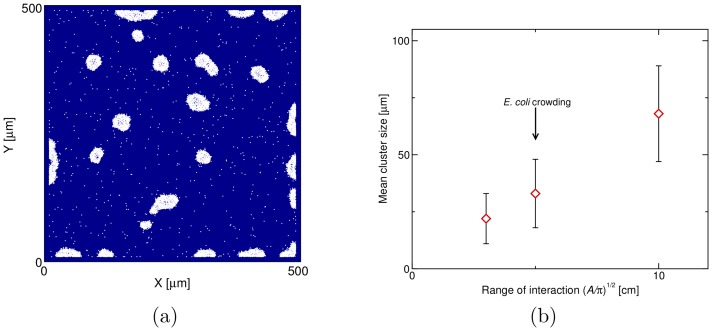
Bacterial clustering induced by a density dependant motility. (**a**) Patterns formed by 2000 bacteria after 3 h (real time) in 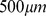 square box (outer walls, no periodic boundary). In this simulation the swimming speed of each cell decreases exponentially with the local density of bacteria within area 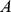 around the cell. The density-dependent slow-down aims at modelling bacterial crowding. Taking a typical size of *E.coli* cells and their flagella, 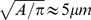, and the bacterial clusters that form are around 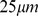 large with irregular shapes. Parameters were 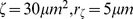. Note that the clusters are unstable towards coarsening, although the coarsening is slower than in the case of chemotactic aggregation in Fig. 1. (**b**) The mean cluster size as a function of the area 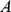 used to define the local density. Increasing 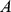 leads to larger clusters, however, a large value of 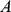 cannot be used to model bacterial crowding.

Fig. 6 explores the dynamics of a bacterial suspension of similar density than the one in Fig. 1A, but with the additional feature that the bacteria regulate their swimming speed according to the local density rather then by sensing chemoattractant. The local density is measured in an area of size 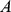 around each cell. If the physical reason behind the slowdown is steric – bacteria slow down where they jam – then it is reasonable to assume that the area 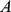 is about the size of a bacterium plus flagellum, i.e. around 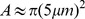, which is the choice made in the simulations in Fig. 6. In this case, we do observe spot formation starting from an initially uniform suspension, however the spot size is much smaller than that of the clusters formed by chemotactic aggregation in Figs. 1 and 2 (compare the spatial scale there with that in Fig. 6). The spot size can in principle be controlled by varying 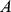 (see Figure 6b). However, a value of 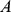 larger than the size of bacteria with flagella cannot represent the crowding mechanism; it rather corresponds to longer-range interactions chemically mediated by, e.g. quorum-sensing interactions. Therefore, by increasing the range of interaction beyond 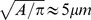, we interpolate between the simple crowding model and the model with sensing chemoattractant, as in Figs. 1 and 2.

Intriguingly, in the experiments in Ref. [8] it was observed that clustering proceeds in a two-step fashion. First chemotactic aggregation proceeds, with a spot size similar to the one in the experiments in Ref. [5]. About 10 hours later, the clusters disassemble to form a “bacterial crystal” made up by smaller spots, about 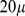
m in diameter – their size and irregular ordering is compatible to those obtained with a sterically induced motility slowdown as in Fig. 6.

## Discussion

In conclusion, we have presented here a microscopic model for pattern formation in suspensions of reproducing and chemotactic bacteria. With respect to previous macroscopic approaches based on mean field approximations, our microscopic model resolves individual bacteria, hence it can account for fluctuations in the bacterial density, as well as for its effects on the onset of pattern formation and on the nature of the patterns.

The underlying microscopic force driving the pattern formation in our model is a self-trapping feedback mechanism which couples an increase in chemoattractant secretion due to, for instance, a local density fluctuation, to further chemotactically driven bacterial accumulation. This accumulation leads to first droplet formation, then coarsening, such that a single bacterial spot is stable in the steady state. In the absence of reproduction, the steady-state position depends on geometry in a nontrivial way (see Fig. 1): if a wall is present the spot is driven there; in a square chamber bacteria eventually accumulate at the corners, while in a setup where a box with a small opening is placed in the middle of the simulation domain, bacteria spontaneously enter into the box and aggregate there. The latter result is in agreement with experimental observations of chemotactic aggregation in microfluidic devices [8].

We next simulated pattern formation of bacterial colonies, set up so as to model experiments in Refs. [4], [5]. Instead of starting from a uniform suspension, we now initialised the simulations with an inoculum of bacteria, from which the bacteria spread outwards. We have mapped the parameters determined from the experimental results [5] in order to model realistic bacterial populations. We considered three cases: bacteria sense (i) only chemoattractant gradients, (ii) only nutrient gradients or (iii) both. In the first case (i) the spread of bacteria resembles a Fisher-wave: this is because bacteria do not move towards source of nutrients, but still they consume it and their reproduction rate depends on its local concentration. Rather than a disc like shape, as is achieved in a standard Fisher wave, the bacteria form a wide spreading ring with immobile spots, which arise due to the previously discussed self-trapping mechanism. This result closely resembles the experiments in Refs. [4], [5]. For the case (ii) when sensing is only towards nutrients, our simulations reproduce the well known results where the bacterial colony forms a single chemotactic ring spreading outwards. Simulations suggest that case (iii), where we assume that bacteria sense both, the nutrients and the chemoattractant, is particularly interesting. Here the results depend on the ratio between the efficiency in the chemotactic sensing of the two chemicals. In particular, we fixed the chemoattractant sensing strength and varied the efficiency of sensing the nutrients. For weak nutrient sensing the bacterial colony forms a pattern with fixed spots, very similar to the one case discussed above without nutrient sensing. The main difference is that now the patterns are more symmetric as their position is guided by the dynamics of the swarming rings. For slightly stronger nutrient sensing the bacteria still form spots, but these are now mobile and chemotactic, as they can glide up a nutrient gradient. The motile bacterial spots re-merge after the initial breakup of the ring. We therefore suggest that it would be worthwhile to design further experiments with bacterial strains where the ratio between chemotactic sensing of nutrient and chemoattractant gradients can be sensed; if such experiments were done, we suggest that one may be able to see a crossover between distinct patterns, both dynamically and in steady state.

We finally considered a simpler model where the swim speed depends on the local bacterial density. This was previously proposed to account for “chemotactic” patterns in *S. typhimirium* [15]. The mechanism through which the patterns form in this case is again due to a self-trapping mechanism, although the slowdown does not need to be chemically controlled. We have assumed an interaction range for the slowdown model which is compatible with sterically suppressed motion (for instance arising when the bacteria jam due to the high local density). In this case, the size of the patterns is orders of magnitude smaller and their shape is more irregular than the one found via chemotactic aggregation in Figs. 2, 4 and 5. However, the patterns obtained are quite akin to the “bacterial crystals” reported in Ref. [8] where they observe formation of small irregular spots of *E. coli* in a micro-fabricated maze, several hours after the primary chemotactic patterns have appeared.

## Materials and Methods

### Basic features of the model

In our work, we model bacteria as two-dimensional hard disks with radius 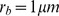, which swim with a constant speed 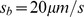. In the absence of chemical gradients, the direction of motion changes due to an effective rotational diffusion 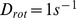, which models tumbling at a realistic rate for *E. coli*. We further assume that the effect of chemotaxis can be included by adding a drift velocity term to the velocity of bacteria, renormalising its magnitude so that total speed remains constant.

The model requires knowledge of how the drift velocity depends on gradients, but this is largely known from the literature. Because we treat tumbling of bacteria implicitly, here we do not simulate chemical reactions or solve memory integrals which control the tumbling rate [18], [21]–[24]. This approach allowed us to write a very fast code: for instance, a simulation of about 30 k bacteria on a single core takes 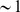 second of CPU time per second of physical time. We also need to simulate diffusion of nutrient and chemoattractant; to this end we set up a finite difference algorithm on an underlying lattice (mesh size 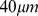): this part of the code takes up only a limited fraction of the total computational time. Using MPI parallelisation on 64 or 256 cores, we simulated real macroscopic systems 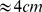 square box with up to 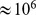 bacteria with the same one-to-one mapping between simulation and physical unit of time.

Our microscopic model, where the chemotactic drift velocity and bacterial diffusion are essentially dialled in directly, has been designed to allow easy comparison with previous continuum mean field models where the bacterial population and concentration of chemicals (nutrients or chemoattractant) evolve according to a set of partial differential equations [7], [8], [15], [25]. With respect to those approaches, importantly, our microscopic model naturally incorporates the effect of noise and density fluctuations in the bacterial colony – the latter can make a difference to pattern formation problem, especially when they can be seen as non-equilibrium phase transitions [15].

In summary, our model is computationally cheap, but at the same time allows us to relax the mean field approximation common to a large part of the literature on bacterial pattern formation. Given the current high performance and parallel computational facilities, the model can deliver realistic microscopic simulations of macroscopic systems like 8 cm Petri dish.

### Tuning chemotactic sensitivity

We now discuss how to modify the drift velocity of a bacterium in response to the gradient of a chemical (either chemoattractant or nutrient in our simulations). Bacteria exhibit two general types of sensing, depending on the environment: (i) absolute gradient sensing where the drift velocity is proportional to gradient of a chemical (here 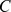); and (ii) relative gradient sensing, (also called logarithmic sensing) where the drift velocity is proportional to 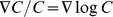.

Guided by the discussions in Refs. [26], [27] based on experiments and explicit modelling of the chemotaxis signalling pathway, we here postulate that at low concentration of chemicals, bacteria sense absolute gradient, while at higher concentrations they sense relative gradient. The drift velocity in our model is thus given by
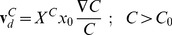
(1)

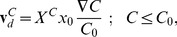
(2)where 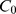 is the crossover concentration between absolute and relative sensing. For our simulations we chose 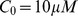 [26]. On the other hand, 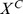 is a dimensionless parameter specifying the chemotactic efficiency and 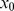 gives the reference drift velocity per gradient, which we take to be 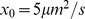, relevant for wild type *E. coli* [27], [28].

For chemoattractant gradients, 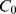 is large enough that only absolute sensing is relevant at realistic bacterial densities. This is motivated by the experiments in Refs. [4], [5], which always measured low chemoattractant concentration (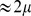
M). Furthermore, if we were to assume logarithmic sensing, 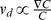, then the intuition suggests that the characteristic dimensionless parameter determining whether cluster formation occurs would be 
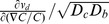
, where 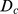 is the diffusion constant of chemicals and 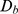 the effective diffusion constant of bacteria. As this parameter is proportional to 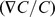, the criterion for clustering formation should not depend on density. Moreover, as pattern formation is fluctuation-driven, it would be more pronounced at low density, where fluctuations are larger, rather than at high density. This is the opposite of what is found experimentally. The experimental trend is, on the other hand, naturally reproduced if bacteria sense absolute chemoattractant gradients, which strengthens the argument in favour of absolute, rather than relative, chemotactic sensing for chemoattractant. For absolute sensing, the dimensionless parameter determining whether clusters form is 
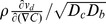
, where 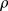 represents the two-dimensional density of bacteria in the system.

When chemotaxis towards both nutrients (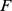) and chemoattractant (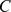) is simulated, the total drift velocity is given by
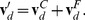
(3)


We additionally cap the drift velocity at some maximum value
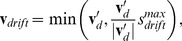
(4)which we take equal to one third of the total bacterial speed. This ensures that after normalisation we get 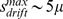
m/s, in line with experiments and simulations (e.g. Refs. [27], [28] give a swim speed of 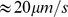 and 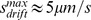).

The bacterial direction in 2D is characterised by a single angle 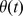. In a rotational diffusion time step this angle changes as:
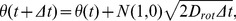
(5)where 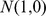 is normally distributed random number with variance 1 and average 0. The resulting velocity after the step is equal to



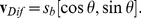
(6)In a complete time step the position of a bacterium, 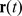 evolves as:
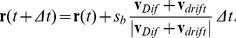
(7)


Note that because of this normalisation the effective diffusion of bacteria is not constant: it decreases when the chemotactic drift is strong. This provides a (small) difference with respect to the partial differential equation models where the diffusion is kept constant [7], [15], [25].

Bacteria divide at a nutrient-dependent rate 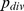, given by the following probability per unit time:
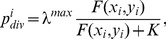
(8)where 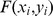 is the local nutrient concentration at the location 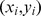 of the bacterium 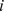 and 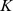 is a “dissociation” constant defining the concentration at which 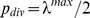. Various experiments with *E. coli* on semisolid substrates (see, e.g. [5]) are compatible with the value 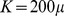
M, which is the value we used in all the simulations. The prefactor 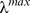 determines the maximum division rate of the bacteria. Given that the lifetime of *E. coli* bacteria under best circumstances is 20 min, the value assumed here is 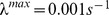. When a bacterium divides, it splits into two disks, slightly displaced along a random angle around the centre of the “parent”. Due to their steric repulsion, the two resulting bacteria will quickly separate from each other. The nutrient consumption is given by a similar equation:
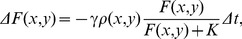
(9)where 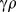 is the maximum nutrient consumption rate and 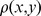 is the local density of bacteria. Chemoattractant production is constant per bacteria, it also spontaneously decays (this is necessary in order to reach a steady state):
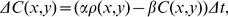
(10)where 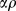 and 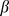 are the production rate and the decay rate of chemoattractant, respectively. The nutrient and chemoattractant molecules diffuse in the medium with the diffusion constant 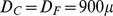
m
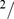
s [28].

### Model parameters

In order to resolve the relevant spatial and temporal scales, in our simulations we have used the appropriate values for the time step 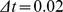
s and for the mesh size to model distribution of chemicals, 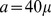
m. We have adopted the well-established values of some of the physical parameters in the model – as explained above in particular a realistic swimming speed 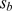 and division rate 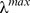 for *E. coli*. The experimentally relevant values of the remaining model parameters 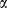, 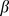 and 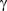, are – to the best of our knowledge – unknown. Moreover, their values depend on various external conditions that may vary significantly from experiment to experiment or in natural environments. However, by carefully analysing the experimental data in [5], we were able to estimate the realistic order of magnitude for each of the three parameters. The decay rate of chemoattractant 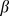 can be estimated from the fact that a steady state in aspartate concentration seems to be reached in a few hours (Figure 2b in [5]), thus a reasonable value is 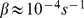. From the same figure we can read that the steady-state concentration of the chemoattractant, which we denote as 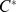, lies in the range 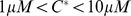. Since the steady-state value corresponds to the ratio between the chemoattractant production and the decay rate, 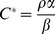, the value of 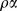 must be in the range 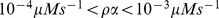. The nutrient intake rate of bacteria 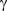 should be much larger, 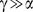, as the bacteria can secrete only a fraction of the chemicals they consume. An estimate from Figure 2c in [5] is that bacteria consume the nutrient with initial concentration 1 mM in about 10 to 30 hours, thus 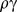 is within 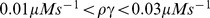.

In our simulations, a typical density of bacteria was 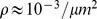, thus the parameter values corresponding to the experimental situation would be 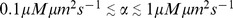, 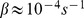 and 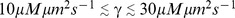. Now, if we used these parameter values, it would require computational times of the order of 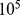 CPU hours, which is not viable. In order to speed up the simulations, we have used a factor of 100 times larger parameter values: 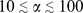, 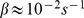 and 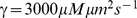 (we have fixed the value of 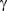 since only the two ratios are important for the morphology of the patterns). In this way we could simulate patterns with around 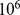 bacteria in 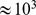 CPU hours of massively parallel simulation runs. Since we have kept the ratio 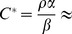 1 to 10 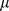
M, thus similar to the experimental one, the simulation and experimental trajectories are in the same sensing regime (i.e. absolute versus relative sensing, the threshold between both is 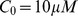) and the emerging steady-state patterns are also similar. We have varied the values of all the uncertain parameters in order to assess the stability of the model. Upon variations within an order of magnitude, we observed qualitatively similar results. The emerging morphology and the characteristic length-scales (i.e., size of the clusters, cluster-cluster distance, size of the rings etc.) depend on both, the ratio 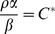 and 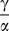 – decreasing the latter results in smaller clusters as can be seen in Fig. 2.
